# Heat production and volatile biosynthesis are linked *via* alternative respiration in *Magnolia denudata* during floral thermogenesis

**DOI:** 10.3389/fpls.2022.955665

**Published:** 2022-10-14

**Authors:** Ruohan Wang, Ling Chen, Yaping Jia, Liya Liu, Liwei Sun, Yujun Liu, Yun Li

**Affiliations:** ^1^ National Engineering Laboratory for Tree Breeding, College of Biological Sciences and Biotechnology, Beijing Forestry University, Beijing, China; ^2^ Key Laboratory for Genetics and Breeding of Forest Trees and Ornamental Plants, Ministry of Education, College of Biological Sciences and Biotechnology, Beijing Forestry University, Beijing, China; ^3^ Institute of Botany, Chinese Academy of Sciences, Beijing, China

**Keywords:** floral thermogenesis, high-throughput sequencing, alternative respiration, cell wall, volatile organic compounds

## Abstract

Floral thermogenesis is coupled with odor emission in known thermogenic plants. It is widely accepted that elevation in floral temperature can help release of volatile organic compounds (VOCs). However, no information is available about whether floral thermogenesis is associated with VOC biosynthesis. Here, we used RNA-Sequencing (RNA-Seq) to draw a gene expression atlas of floral thermogenesis in *Magnolia denudata* and captured an upregulation of *Alternative Oxidase* (*AOX*) during floral thermogenesis. Western blot analyses also suggested upregulation of AOX during floral thermogenesis. Moreover, oxygen consumption analyses revealed increased activity of the AOX respiration pathway during floral thermogenesis. Using HPLC analyses, we further found that increased AOX respiration substantially promoted production of citric acid by 1.35 folds, which provided fundamental metabolite skeletons for biosynthesis of VOCs. RNA-Seq also showed upregulation of genes regulating lignin catabolism, which was in agreement with *in situ* Raman chemical imaging of lignin. Taken together, our results suggest the central role of *AOX* by coupling heat production and VOC biosynthesis in floral thermogenesis of *M. denudata*.

## Introduction

Animal-like heat production has been observed in some plant taxa. Since the first description of floral thermogenesis in arum lily (*Arum*, Araceae) by Lamark (1778) there have been an increasing number of reports on plant thermogenesis (Nagy et al., 1972; Seymour et al., 2003; Lamprecht et al., 2006; de Bruyn et al., 2015). Heat produced spontaneously by thermogenic plants can maintain flowers at higher temperatures than the ambient environment (Li and Huang, 2009; Miller et al., 2011; Terry et al., 2014). *Philodendron selloum* spadices can even heat themselves to 38 - 45°C when the ambient temperature is near freezing (Nagy et al., 1972). In recent decades, floral thermogenesis has generally been considered a heat reward for insect pollinators to increase pollination (Cooley, 1995; Seymour et al., 2003; Rands and Whitney, 2008);. Apart from direct heat rewards to insects, it has been increasingly realized that floral thermogenesis is also a critical life history strategy that contributes multiple biological functions to plants. For example, it was recently demonstrated that floral thermogenesis can promote odor emission in *Magnolia sprengeri* and *Nelumbo lutea*, which might help attract insect pollinators (Wang et al., 2014; Wickett et al., 2014; Hernández-Vera et al., 2021). Moreover, in Asian sacred lotus (*N. nucifera*), a constant floral temperature maintained *via* thermogenesis facilitates fertilization (Terry et al., 2014). Furthermore, heat produced during floral thermogenesis is retained in floral chambers in some species, which can also promote pollen development (Seymour et al., 2009a; Seymour et al., 2009b; Liu et al., 2017).

The identification of multi-faceted biological functions of floral thermogenesis has prompted scientists to explore the underlying regulatory mechanisms. Through a series of elegantly designed experiments, Seymour et al. demonstrated that floral thermogenesis is not a by-product of respiratory activities, but instead involves complex mechanisms that govern this biological process (Seymour et al., 2010; Terry et al., 2016). Since the identification of *Alternative Oxidase* (*AOX*) as the key component of cyanide-resistant respiration which dissipates energy as heat in plants, it has been presumed that AOX is a regulator of floral thermogenesis (Rhoads and McIntosh, 1991; Borecky and Vercesi, 2005). Soon after the discovery that *Uncoupling Proteins* (*UCPs*) were responsible for non-shivering thermogenesis in mammals, *UCPs* were also isolated from mitochondria of some thermogenic plants (Vercesi et al., 1995; Ito, 1999). These reports raised the possibility that *UCPs* might also be involved in the regulation of plant thermogenesis. Regardless of these important findings, it remains unknown how plants manipulate the process of floral thermogenesis.

Increasing evidence suggests that more molecules besides *AOX* and *UCPs* are involved in thermogenesis (Chatterjee and Perrimon, 2017; Moraru et al., 2017; Li et al., 2020). Recently, it was reported that *Thyroid Adenoma Associated* (*THADA*) and *Sarcolipin* can also trigger non-shivering thermogenesis in *Drosophila* by uncoupling ATP hydrolysis from the pumping of Ca^2+^ into the endoplasmic reticulum (Maurya et al., 2015; Moraru et al., 2017). By analyzing expressed sequence tags using super serial analyses of gene expression (superSAGE), Ito-Inaba et al. (2012) found that vacuolar metabolic pathways may also be involved in regulating floral thermogenesis of skunk cabbage (*Symplocarpus renifolius*). Evidence has also suggested that photosynthetic electron transport activities might play a role in energy supply during floral thermogenesis (Liu et al., 2015). In this context, the overall mechanisms behind the regulation of floral thermogenesis remain elusive. Emerging omics technologies are enabling comprehensive analyses of regulatory networks of multiple biological processes in various organisms. Transcriptome sequencing is a powerful tool for unbiased profiling of gene expression landscapes to study complex biological processes (Grabherr et al., 2011).

The early diverged angiosperms *Magnolia* (Magnoliaceae) are thermogenic during anthesis (Dieringer et al., 1999; Gottsberger et al., 2012; Wickett et al., 2014). Considering that heat is not evenly produced through a flower (Seymour et al., 2010; Zhang et al., 2015), it is necessary to sample heat-producing tissues precisely to pinpoint the regulatory events associated with floral thermogenesis. Recently, we established a customized high-resolution infrared thermal imaging system with a spatial resolution of 42 µm and a temperature resolution of 0.02°C, which allowed precise detection of heat-producing tissues in *Magnolia* flowers (Wickett et al., 2014; Liu et al., 2015). Using this thermal imaging system, we have demonstrated that gynoecium is the main heat-producing tissue in *Magnolia denudata* flowers (Wang et al., 2013). Based on accurate sampling of heat-producing tissues, we here combined transcriptome sequencing with multiple approaches, including chemical imaging and physiology, to deepen our understanding of the regulatory framework of floral thermogenesis in *M. denudata*.

## Materials and methods

### Plant materials and RNA isolation

**Figure 1:**
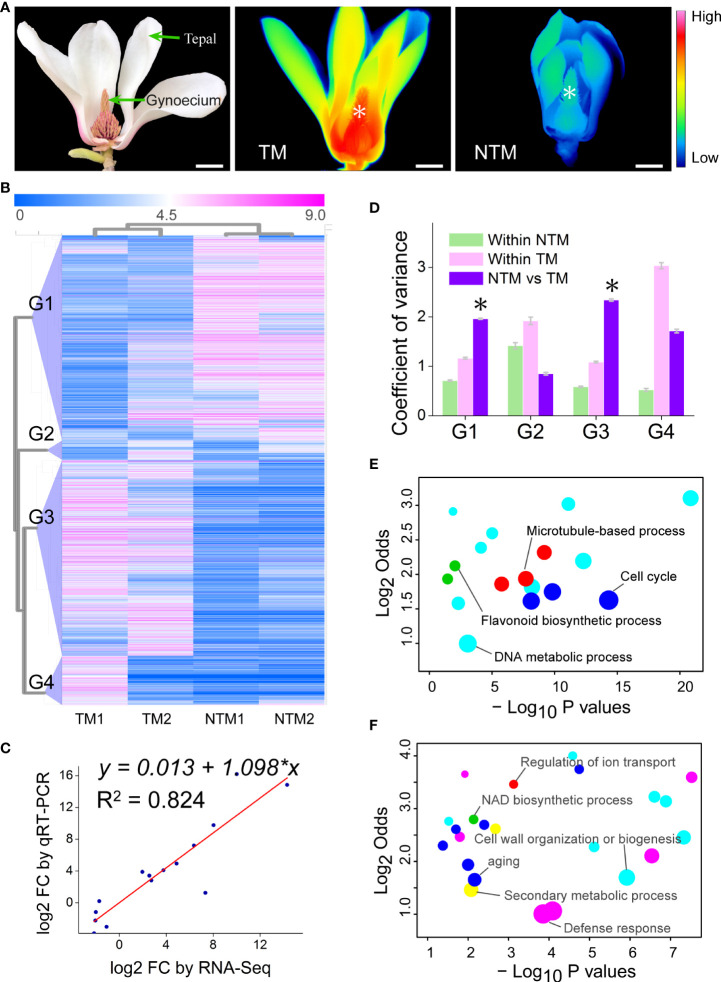
Transcriptomic changes in the gynoecium during floral thermogenesis of *Magnolia denudata*. **(A)** A photograph (left) of the *M. denudata* flower and infrared images of the flower at TM (middle) and NTM (right) stages. Temperatures in the infrared images are color coded, with red colors indicating higher temperatures and blue colors lower temperatures. The asterisks in the infrared images indicate the major thermogenic tissue, namely gynoecium. Scale bars, 3 cm. **(B)** A heat map depicting DEGs between TM and NTM gynoecium. Data of expression levels of the DEGs were log_2_transformed and coded in color, with magenta indicating higher expression levels and blue lower levels. The DEGs were clustered into four groups (G1-G4) by hierarchical clustering analyses. **(C)** The FCs showed close correlationship (Pearson correlation coefficient = 0.908, p = 2.887×10^-5^) between RNA-Seq and qRT-PCR analyses for the 15 arbitrarily chosen genes. **(D)** Variation in expression levels of the four groups of DEGs. The asterisks indicate that variation in expression levels were significantly (p < 0.05) higher between TM and NTM libraries than those within two biological replicates of TM or NTM for DEGs. **(E, F)** Enriched GO BP terms for DEGs of Groups 1 **(D)** and 3 **(E)**. GO terms with similar biological processes were indicated in the same color. Sizes of the circles indicate numbers of DEGs annotated to the certain GO terms; GO terms with the highest number of DEGs in the same color were indicated as the representative ones and labeled. The detailed GO terms are given in **Supplementary Table S6**.

Flower samples were collected from *Magnolia denudata* Desr. (Magnoliaceae) trees at the campus of Beijing Forestry University (40°00′03″ N, 116°20′25″ E, a.s.l., 68 m). We used a modified infrared thermography radiometer (TiX660; Fluke Co., Everett, MA, USA) to guide the sampling of flowers at non-thermogenic (NTM) stages and thermogenic (TM) for the current study (**Figure 1A**). This radiometer can be used to perform real-time measurement of gynoecium temperature and ambient air temperature (Wickett et al., 2014). Only flowers with temperatures 4 - 6°C above the environmental temperature in gynoecium were identified as TM, and flowers with temperatures equivalent to that of the environment were collected as NTM samples. Gynoecium from TM and NTM flowers was cut, immediately frozen in liquid nitrogen, and stored at -80°C until use.

Total RNA was extracted from TM and NTM gynoecium (with two biological replicates for each) using Trizol reagent (Invitrogen, Carlsbad, CA, USA) following the manufacturer’s procedure. Total RNA quantity and purity were analyzed using Bioanalyzer 2100 and RNA 6000 Nano LabChip Kit (Agilent, Santa Clara, CA, USA) with RNA integrity number >7.0. High-quality RNA from the same biological replicate was split for the construction of the RNA-Seq library.

### mRNA library construction and RNA-Seq analyses

Two biological replicates were prepared for each of the TM and mRNA libraries. Following purification and mRNA fragmentation, cDNA libraries were constructed in accordance with the protocol for the Illumina RNA ligation-based method (Illumina, San Diego, CA, USA). Sequencing was performed by LC Science (Hangzhou, China) on an Illumina HiSeq 2000 platform. Raw data containing adaptor sequences, tags with low-quality sequences, and unknown nucleotides N were filtered out and clean reads subjected to quality assessment. Clean reads of the four libraries were pooled for *de novo* assembly of a global transcriptome *via* Trinity (Grabherr et al., 2011). Functional annotation of transcriptome genes was performed using the unigene sequences as queries for BLAST analyses against online databases, including the NCBI NR protein, Swiss-Prot, Pfam, KOG, and KEGG databases. Gene expression levels were calculated by mapping the clean reads of each biological replicate (library) to the global transcriptome using Bowtie 2 (Langmead and Salzberg, 2012) and normalized to the RPKM number. GO enrichment was performed using the agriGO analyses tools (Du et al., 2010) and the enriched GO terms were visualized *via* REVIGO (Supek et al., 2011) and R scripts (R Development Core Team, 2012).

### Western blot analyses

To quantify the abundance of AOX and UCP proteins, we first isolated mitochondria from *M. denudata* flower tissues and then conducted western blot analyses. Briefly, mitochondria were isolated from fresh tissues in accordance with a previous study (Day et al., 1985), with minor modification. Total protein was extracted from isolated mitochondria in lysis buffer. After cleaning of lysates at 1300×*g* for 10 min, supernatants were quantified for protein content. Equal amounts of protein samples were separated by SDS-PAGE and immunoblotted with antibodies against AOX (AS10 699; Agrisera,Vännäs, Sweden) and UCP1/2(AS12 1850; Agrisera).

### qRT-PCR

Total RNA was extracted from *M. denudata* flower samples using RNAiso Plus (Takara, Dalian, China) following the manufacturer’s protocol. First-strand cDNA was synthesized using Transcript One-Step gDNA Removal and cDNA Synthesis SuperMix (TianGen Biotech, Beijing, China). qRT-PCR reactions were conducted in triplicate on an ABI 7500 real-time PCR detection system (Applied Biosystems) using SYBR Green qRT-PCR Mix (Toyobo, Osaka, Japan). Relative gene expression levels were calculated using the 2^ΔΔCt^ method (Livak and Schmittgen, 2001), with *GAPDH* used as an internal reference for mRNAs. Gene-specific primers for PCR assays are listed in **Supplementary Table S1**.

### *In vivo* respiration measurement

Discs of 5 mm in diameter (*ca.* 1 mm thick) were cut from fresh leaf, gynoecium, and tepal tissues and measured for their oxygen consumption rate (OCR) using XF24-3 Extracellular Flux Analyzer (Seahorse Bioscience, Billerica, MA, USA). OCR was measured following a published protocol (Ji et al., 2015). Briefly, fresh samples were fixed in 24-well XF measuring plates and incubated in respiration buffer (10 mM HEPES, 10 mM MES, 2 mM CaCl_2_). Initial OCR was measured for five cycles (mixing, 3 min; waiting, 1 min; measuring, 2 min) before adding respiration inhibitors. After the cytC respiration inhibitor (NaN_3_, 20 mM) had been added, real-time OCR was measured for another eight cycles until respiration remained stable. Then an AOX respiration inhibitor (salicylhydroxamic acid, 5 mM) was added, and real-time OCR measured for six cycles until a steady level of respiration was reached. The measured OCR was normalized by sample weight.

### Measurements of citric acid

Extraction and analyses of citric acid was conducted as described by Li et al. (2016). 500 mg of NTM and TM gynoecium was ground to powder in liquid nitrogen and homogenized in 3 ml methanol (40%) by ultrasound (BILON22-600B, Bilang, Shanghai, China) for 30 min. Then homogenate was centrifuged at 12,000 ×*g* for 10 min and the supernatant filtered with Φ 0.22 µm nylon filter (Jinteng, Tianjin, China). The filtered solution was transferred into a vial and supplemented with 40% methanol to 2mL. The measurement of citric acid was performed on high-performance liquid chromatography (HPLC) (Shimadu, Kyoto, Japan) with a Diamonsil C18 column (250 × 4.6 mm, particle size 5 µm, Dikma, Beijing, China). The flow rate was 400µL min^-1^ using miliQ water (with 0.1% H_3_PO_4_, phase A) and methanol (phase B) as the binary mobile phase. A UV detector was set at 214 nm for the detection of citric acid.

### Confocal raman microscopy

For confocal Raman microscopy, gynoecium of *M. denudata* was collected, immediately frozen in liquid nitrogen, and then stored at -20°C. Frozen sections (12 μm thick) were cut on a Leica 1950 frozen microtome (Leica, Wetzlar, Germany) and used for label-free *in situ* Raman imaging. Raman spectra were acquired on a confocal Raman microscope (LabRam Xplora; Horiba Jobin Yvon, Longjumeau, France) equipped with an Olympus BX51objective (100×, oil, NA=1.40; Olympus, Tokyo, Japan). A linear polarized laser (λ= 532 nm) was focused with a diffraction-limited spot size of 1.22 λ/NA and the Raman light detected by an air-cooled front-illuminated spectroscopic CCD behind a grating (2,400 grooves mm^-1^) spectrograph with a resolution of 1.5 cm^-1^. The laser power on samples was approximately 8 mW. For mapping, 0.5 μm steps were chosen and every pixel corresponds to one scan.

Spectral analyses and chemical imaging were performed using Labspec software (Horiba Jobin Yvon), as previously described (Ma et al., 2013). Briefly, cosmic rays were removed and sum filters were applied to integrate defined regions in the acquired spectra. Within a chosen area, the sum filter was used to calculate the intensities. Average spectra were calculated and baseline corrected prior to further analyses of signal intensities. The integrated intensity of the lignin band at 1500-1700 cm^-1^ was used for the semi-quantitative analyses of lignin content.

## Results

### *De novo* assembly of the transcriptome and gene annotation

Guided by infrared thermal imaging, we precisely sampled TM and NTM gynoecium and built four transcriptomic libraries (**Figure 1A**). High-throughput RNA-Seq generated a total of 40,936,295 raw reads in the four libraries (**Supplementary Table S2**). After cutting adaptors followed by the removal of junk reads, we obtained 40,821,911 valid reads (**Supplementary Table S3**), accounting for 99.72% of all raw reads, which suggested the high quality of the sequencing data. Valid reads of the four libraries were pooled and *de novo* assembled *via* Trinity (Grabherr et al., 2011), producing 73,962 transcripts (**Supplementary Table S4**). Then the transcripts were clustered with >95% similarity, which generated a transcriptome with 61,212 unigenes.

Transcriptomic unigenes were annotated using BLASTX searches against the NCBI non-redundant protein sequences (NR), Swiss-Prot, and Pfam databases. A total of 35,033 unigenes were matched to known genes in the NR database with an E-value of < 10^-5^ accounting for 57.23% of total unigenes (**Supplementary Table S5**). Similarly, 21,679 (35.42%) unigenes were identified in Swiss-Prot and 25,595 (41.81%) in the Pfam database. To further annotate the transcriptome unigenes, we used Gene Ontology (GO), Eukaryotic Ortholog Groups (KOG), and Kyoto Encyclopedia of Genes and Genomes (KEGG) assignments to classify their functions. Based on sequence similarity, 19,045 (31.11%) unigenes were annotated in the GO database (http://geneontology.org/). A total of 19,069 (31.15%) unigenes were categorized into 25 KOG functional groups, among which “general function prediction only” represented the largest group, followed by “signal transduction mechanisms”, “posttranslational modification, protein turnover, chaperones”, and “transcription” (**Supplementary Figure S1**). There were 14,194 (23.19%) unigenes assigned to 267 KEGG pathways, of which “purine metabolism”, “starch and sucrose metabolism”, “ribosome”, and “ubiquitin mediated proteolysis” were the most highly represented pathways.

### Transcriptomic atlas during floral thermogenesis

To investigate the change in gene expression programs during floral thermogenesis, we globally profiled mRNA levels in both TM and NTM gynoecium (**Figure 1A**). Genes with an expression level of ‗1 Reads per Kilobase of exon model per Million mapped reads (RPKM) in at least one library were considered detectable. By adopting the criteria of log_2_foldchange (FC) ‗2 and p < 0.05 (p values were adjusted by the false discovery rate for all differential gene expression analyses of sequencing data), we found 4,342 differentially expressed genes (DEGs) between TM and NTM samples (**Figure 1B**). Expression levels of DEGs were validated using qRT-PCR for 15 arbitrarily chosen genes. The FC revealed by qRT-PCR were closely correlated to those by RNA-Seq (**Figure 1C**), supporting the reliability of the high throughput data.

Principal component analysis (PCA) was performed to cluster the four transcriptome libraries based on expression patterns of the DEGs. The first two PCs explained 85.20% of the total variance in gene expression patterns and showed a substantial distinction between TM and NTM libraries (**Supplementary Figure S2**). Hierarchical clustering analyses also showed clear similarities between two biological replicates with the same physiological status (**Figure 1B**). These results suggest high reproducibility of our biological replicates. Hierarchical clustering analyses indicated that the DEGs could be categorized into four groups showing differential expression patterns (**Figure 1B**). DEGs in Groups 1 and 3 had lower variation in expression levels between biological replicates than between TM and NTM samples (**Figure 1D**). In the remaining two groups (Groups 2 and 4), DEGs showed higher variation in expression levels between biological replicates than between TM and NTM flowers (**Figure 1D**). For higher confidence, DEGs in Groups 1 and 3 (3,708 in total, accounting for 85.40% of all DEGs), rather than in the other two groups, were used to characterize transcriptomic change between TM and NTM samples.

We further performed GO enrichment analyses to investigate the biological functions of these thermogenesis-related DEGs (DEGs in Groups1 and 3). There were 1,896 DEGs downregulated (Group 1) during floral thermogenesis (lower expression levels in TM than in NTM samples); GO enrichment analyses revealed that these DEGs were enriched in GO biological process (BP) terms related to DNA metabolic process, flavonoid biosynthetic process, cell cycle, and microtubule-based process (**Figure 1E**, **Supplementary Table S6**). DEGs that were upregulated (Group 3, 1,812 genes) during floral thermogenesis were enriched in GO BP terms associated with defense response, secondary metabolic process, cell wall organization and biogenesis, aging, NAD biosynthetic process, and regulation of ion transport (**Figure 1F**, **Supplementary Table S6**).

### Respiration and heat production during floral thermogenesis

**Figure 2:**
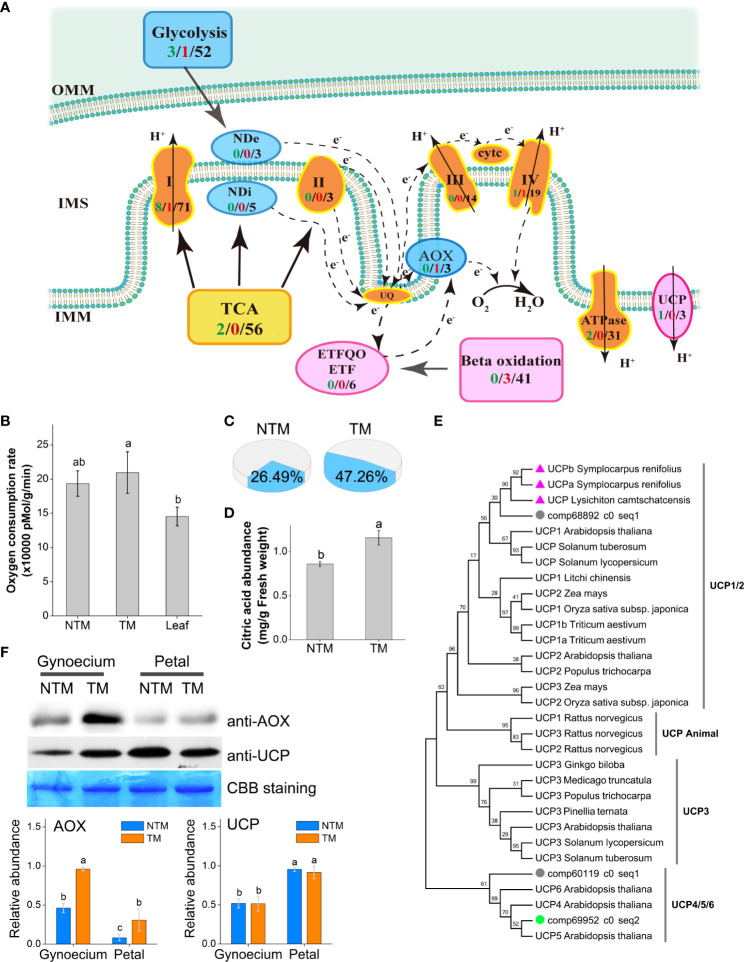
Involvement of the AOX pathway and the UCP in floral thermogenesis of *M. denudata*. **(A)** DEGs involved in the ETC. The arrows indicate the directions of electron transport. The COX respiration pathway is indicated in bright brown, AOX pathway in blue, and lipid metabolism and the UCP in magenta. The numbers in black present the number of unigenes annotated to the certain functional group, the green numbers present downregulated DEGs and the red numbers present the upregulated DEGs during floral thermogenesis. **(B)** Oxygen consumption rates of NTM and TM gynoecium and the leaf. **(C)** Proportion of the AOX pathway in total respiration activity in NTM and TM gynoecium. **(D)** Citric acid abundance in NTM and TM gynoecium. **(E)** Phylogenetic analysis of *UCP*s in different species by Maximum Likelihood method. The bootstrap consensus tree inferred from 1000 replicates is taken to represent the taxa analyzed. The percentages of replicate trees in which the associated taxa clustered together are shown above the branches. The triangles indicate *UCP*s from thermogenic species (excluding *M. denudata*) and the circles represent *UCP*s of *M. denudata*. The green circle shows the *UCP* gene downregulated during floral thermogenesis of *M. denudata*. **(F)** Western blot analyses of AOX and UCP. The Coomassie Brilliant Blue (CBB) staining is shown as a protein loading control. The statistic results of protein abundance were obtained from five biological replicates for AOX and three for UCP. OMM, the outer membrane of the mitochondrion; IMM, the inner membrane; IMS, intermembrane space; ND_e_, external NAD(P)H dehydrogenase; ND_i_, internal NAD(P)H dehydrogenase; CytC, cytochrome C. Data in the bars are means ± standard errors. The same lowercase letters indicate no significant difference (p>0.05) between the samples.

Because floral thermogenesis is usually coupled with intensive respiration in thermogenic plants (Wang et al., 2013), we examined the dynamics of gene expression related to cellular respiration in the four libraries. In total, we detected 56 unigenes encoding key enzymes regulating tricarboxylic acid (TCA) cycling and 52 regulating glycolysis, which substantially provides electrons to the mitochondrial electron transport chain (ETC) (**Figure 2A**). Moreover, we found 138 unigenes regulating the cytochrome oxidation (COX) pathway of ETC, including 71, 3, 14, 19, and 31 genes encoding subunits of complexes I, II, III, IV, and V (ATPase) of ETC, respectively. Meanwhile, we found 11 genes regulating the AOX pathway of ETC, including three, five, and three genes encoding AOX and internal and external NADH dehydrogenases, respectively. Differential gene expression analyses revealed 21 DEGs (8.08%) between TM and NTM samples among the 260 genes involved in mitochondrial respiration (**Figure 2A**). It was interesting to note that 17 of the 24 DEGs were downregulated (clustered in Group 1 DEGs as described above) during the process of floral thermogenesis, most of which were components of Complexes I of the COX pathway (**Figure 2A**). Meanwhile, one unigene (comp72795_c0_seq1) encoding AOX was upregulated during floral thermogenesis (**Figure 2A**).

The respiration activity of *M. denudata* flowers was experimentally determined by measuring the oxygen consumption rate (OCR) during thermogenesis. The NTM gynoecium had an OCR of 193,601 ± 18,554 pMol min^-1^ g^-1^ fresh weight, which was not significantly different from that of the non-thermogenic tissue, namely, the leaf (**Figure 2B**). Although the OCR of TM gynoecium (222,358 ± 30,382 pMol min^-1^ g^-1^ fresh weight) was not significantly different (p >0.05) from that of NTM gynoecium, discrimination analyses showed a significant (p<0.05) increase in the proportion of OCR by the AOX pathway (**Figure 2C**). The AOX pathway contributed to 26.49 ± 8.93% of total OCR in NTM gynoecium, which was significantly (p<0.05) increased to 47.26 ± 7.83% in TM gynoecium (**Figure 2C**). Considering the potential influence of AOX respiration on citric acid, we also measured citric acid content in the gynoecium and detected a citric acid abundance of 1.16 ± 0.08 mg g^-1^ in TM gynoecium, which was significantly (p < 0.05) higher than that of the NTM gynoeciem (0.86 ± 0.03 mg g^-1^) (**Figure 2D**).

Besides AOX, UCPs are another group of regulators of heat production in plants (Krauss et al., 2005; Vercesi et al., 2006). We detected three unigenes encoding UCPs in *M. denudata*. Phylogenetic analyses revealed that comp68892_c0_seq1 is a homolog of *UCP1* of other plants (**Figure 2E**). In the phylogenetic tree, it was closer to homologs of floral thermogenic plants than those of non-thermogenic ones. There were also two unigenes, comp60119_c0_seq1 and comp69952_c0_seq2, encoding homologs of *UCP4/5/6* of *Arabidopsis*. Among these *UCP* genes, only comp69952_c0_seq2 showed significant differential expression (log_2_FC ‗ 2 and p < 0.05) between TM and NTM samples, with higher expression levels in NTM gynoecium. For further investigation of the potential regulatory roles of AOX and UCPs in floral thermogenesis of *M. denudata*, we analyzed the abundance of AOX and UCP proteins during floral thermogenesis using western blotting. AOX protein abundance was significantly (p < 0.05) higher at the TM than at the NTM stage in both gynoecium and tepals (**Figure 2F**). Notably, the highest level of AOX protein abundance among all the samples tested was found in TM gynoecium, which was 2.5-fold higher (p < 0.05) than the NTM gynoecium (**Figure 2F**). In contrast to AOX, UCPs showed higher protein abundance in tepals than in gynoecium, and there was no significant difference (p > 0.05) of UCP abundance between the NTM and the TM gynoecium (**Figure 2F**).

### Cell wall dynamics during floral thermogenesis

**Figure 3:**
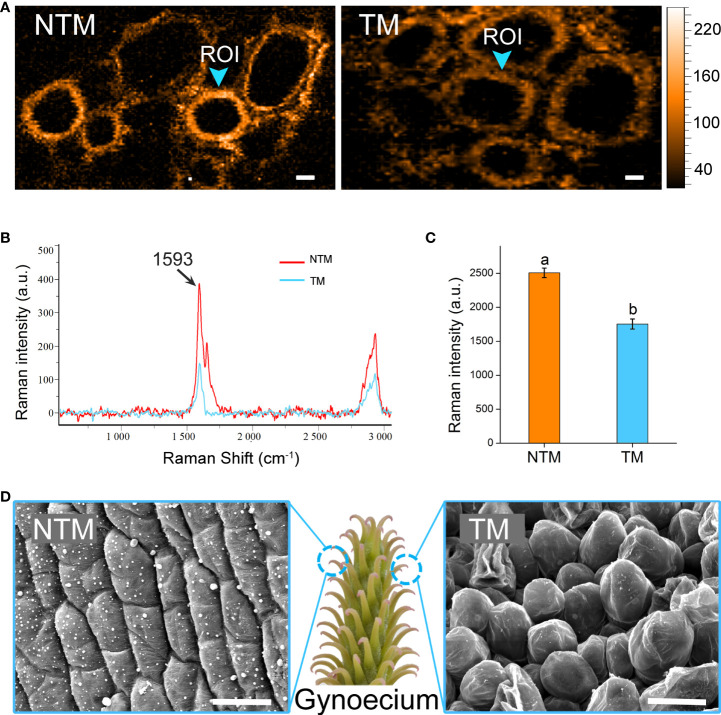
Lignin dynamics and cytological changes during floral thermogenesis of *M. denudata*. **(A)** Raman images of gynoecium cells at the NTM and TM stages. The Raman images are captured by integrating over characteristic lignin band of 1500-1700 cm^-1^. The Raman signal intensity is color coded. Scale bar, 5μm. **(B)** Average Raman spectra acquired from the regions of interest (ROI) from Panel **(A)**. **(C)** Comparison of Raman signal intensities between cell walls of NTM and TM gynoecium at the Raman band most representative of lignin, 1593 cm^-1^. The lowercase letters indicate significant difference (p>0.05) between the samples. Data of the bars are means ± standard errors. **(D)** Scanning electron microscopic images of NTM and TM gynoecium cells. Scale bar, 20μm.

Intriguingly, the DEGs upregulated during floral thermogenesis were enriched in GO BP terms related to cell wall organization and lignin catabolism. We analyzed lignin dynamics experimentally in gynoecium during floral thermogenesis. Newly developed confocal Raman microscopy enabled non-invasive and *in situ* analyses of lignin content in plant cells. By integrating Raman signals of 1500 – 1700 cm^-1^ which are marker bands of lignin (Saar et al., 2010; Ding et al., 2012), we mapped the lignin distribution in gynoecium cells at TM and NTM stages (**Figures 3A, B**). Raman signal intensity for lignin was significantly (p < 0.05) lower in TM than in NTM gynoecium cells (**Figure 3C**), which was in line with the transcriptome sequencing data that DEGs associated with lignin catabolism were upregulated during floral thermogenesis. In addition to the lignin content, we also found that there were some cytological changes. For example, compared with those in the NTM stage, gynoecium cells became “inflated” at the TM stage, and there was some visible intercellular space (**Figure 3D**).

## Discussion

The coexistence of AOX and UCPs in plant mitochondria has raised the question of why plants need two energy-dissipating systems, which has also led to ongoing debate about whether AOX or UCPs are the key regulator in floral thermogenesis (Meeuse and Raskin, 1988; Onda et al., 2007; Miller et al., 2011). In some plants, AOX shows increased expression at the mRNA and/or protein levels during thermogenesis (Onda et al., 2007; Miller et al., 2011). Moreover, AOX has been identified in plants but not in animals. These lines of evidence suggest that AOX plays key regulatory roles in floral thermogenesis. On the other hand, UCPs were differentially expressed in plants between thermogenic and non-thermogenic tissues, which indicated that UCPs play regulatory roles in floral thermogenesis (Ito-Inaba et al., 2008a; Ito-Inaba et al., 2008b). Regarding this puzzle, it was proposed that plants may activate different energy-dissipating systems depending upon the respiratory substrates; for example, starch fuels AOX- whereas lipids fuel UCP-mediated floral thermogenesis (Ito and Seymour, 2005; Watling et al., 2006). Here, we found increased mRNA levels of AOX but not UCPs in the thermogenic tissue of *M. denudata* flowers by transcriptome sequencing, which might suggest that AOX is the regulator. However, the transcriptome data also revealed upregulation of genes involved in lipid metabolism, which might provide respiratory fuel for UCP-mediated thermogenesis. Thus, it might be unreliable to judge whether AOX or UCPs are the key regulator of floral thermogenesis based solely on their expression levels.

A combination of transcriptome sequencing and experimental analyses provided multiple perspectives to dissect the energy-supplying components of floral thermogenesis in *M. denudata*. In line with transcriptome data, western blotting analyses suggested that AOX showed higher protein abundance during thermogenesis in gynoecium (the major thermogenic tissue of *M. denudata* flowers). Although higher protein abundance of UCPs was also detected in gynoecium at the thermogenic than at the non-thermogenic stage, the abundance was notably lower in gynoecium than in the non-thermogenic tissue tepals. Thus, it would be more likely that AOX is responsible for floral thermogenesis in *M. denudata*. The correspondence of AOX but not UCP protein levels with thermogenic activity in the current study is in accordance with previous reports on thermogenic flowers of sacred lotus and *P. bipinnatifidum* (Grant et al., 2008; Miller et al., 2011). Furthermore, oxygen consumption by the AOX pathway was markedly enhanced during floral thermogenesis, which experimentally demonstrated the contribution of AOX to the floral thermogenesis of *M. denudata* (**Figure 2C**). Taking these findings together, AOX is more prone to playing a major role than UCPs in regulating the floral thermogenesis of *M. denudata*. Our findings also suggest the unreliability of inferring the regulatory roles of AOX and UCPs in floral thermogenesis based on respiratory substrates alone.

Intriguingly, total OCR did not show a significant increase during floral thermogenesis in *M. denudata*, which is different from the tight coupling of increased respiration and thermogenic activity in some flowers (Seymour et al., 2010; Seymour et al., 2015). Thus, increased respiration by the AOX pathway, rather than total respiration, can also lead to floral thermogenesis. It has been well summarized that the AOX pathway can function to increase flux rate through the TCA cycle and thus producing key carbon skeletons required for plant metabolism, when the activity of the COX pathway is depressed (Rhoads and McIntosh, 1991; Sweetlove et al., 2010). Respiration of AOX pathway exhibited considerably increased activity during floral thermogenesis in all reports, regardless of the COX pathway (Grant et al., 2008; Miller et al., 2011). Thus, we inferred that the AOX pathway not only functions as an “energy dissipater”, but might also play important roles in supplying crucial metabolite skeletons during floral thermogenesis. Because the TCA cycle intermediate citrate is a fundamental substrate for the mevalonate pathway, which produces various terpenoids as plant VOCs (Sweetlove et al., 2010; Onda et al., 2015), increased activity of AOX pathway respiration may also be involved in floral VOC biosynthesis. Especially, the mevalonate pathway terpenes, including perillene, β-pinene, myrcene, limonene, and α-terpinene, are the major VOC compounds in *M. denudata* (Yasukawa et al., 1992). Recently, it has been reported that a significantly higher level citrate was accumulated in thermogenic *M. denudata* flowers in camparison with non-thermogenic *Magnolia liliiflora* flowers (Park et al., 2018). These findings suggest a link between AOX and VOC biosynthesis during floral thermogenesis. This inference is in agreement with the fact that floral thermogenesis has never been recorded in flowers absent of odor (Meeuse and Raskin, 1988).

Besides the changes in energy-supplying components, our results also revealed dramatic cell wall dynamics during floral thermogenesis. It has been widely recognized that floral thermogenesis can help advertise pollinators by enhancing floral odor emission (Terry et al., 2014; Dieringer et al., 2014; Pereira et al., 2014). Generally, plant VOCs are assumed to be emitted through biological barriers into the air by passive diffusion (Martin et al., 2000; Niinemets et al., 2010). Based on Fick’s first law, floral thermogenesis could thus promote the diffusion process *via* heating the interface directly. Because cell wall is a cellular barrier during plant VOC emission, we should not neglect the influence of cell wall properties on the emission process. Recently, it has been asserted that non-diffusion-related biological mechanisms should be involved in the emission of plant VOCs, which involves directional transport of VOC molecules from the lipophilic plasma membrane to the hydrophilic cell wall mediated by small carrier proteins (McFarlane et al., 2010; Muhlemann et al., 2014; McFarlane et al., 2014; Widhalm et al., 2015). Although it is still unclear how the emission of VOCs is controlled by plants, these findings suggest a possible influence of cell wall properties on this process. Here, we found some morphological changes in cells, which created intercellular spaces during floral thermogenesis. A similar phenomenon was also reported in the thermogenic flowers of *A. maculatum* (Bermadinger-Stabentheiner and Stabentheiner, 1995). These structural changes may also contribute to enhanced VOC emission during floral thermogenesis.

## Conclusion

**Figure 4:**
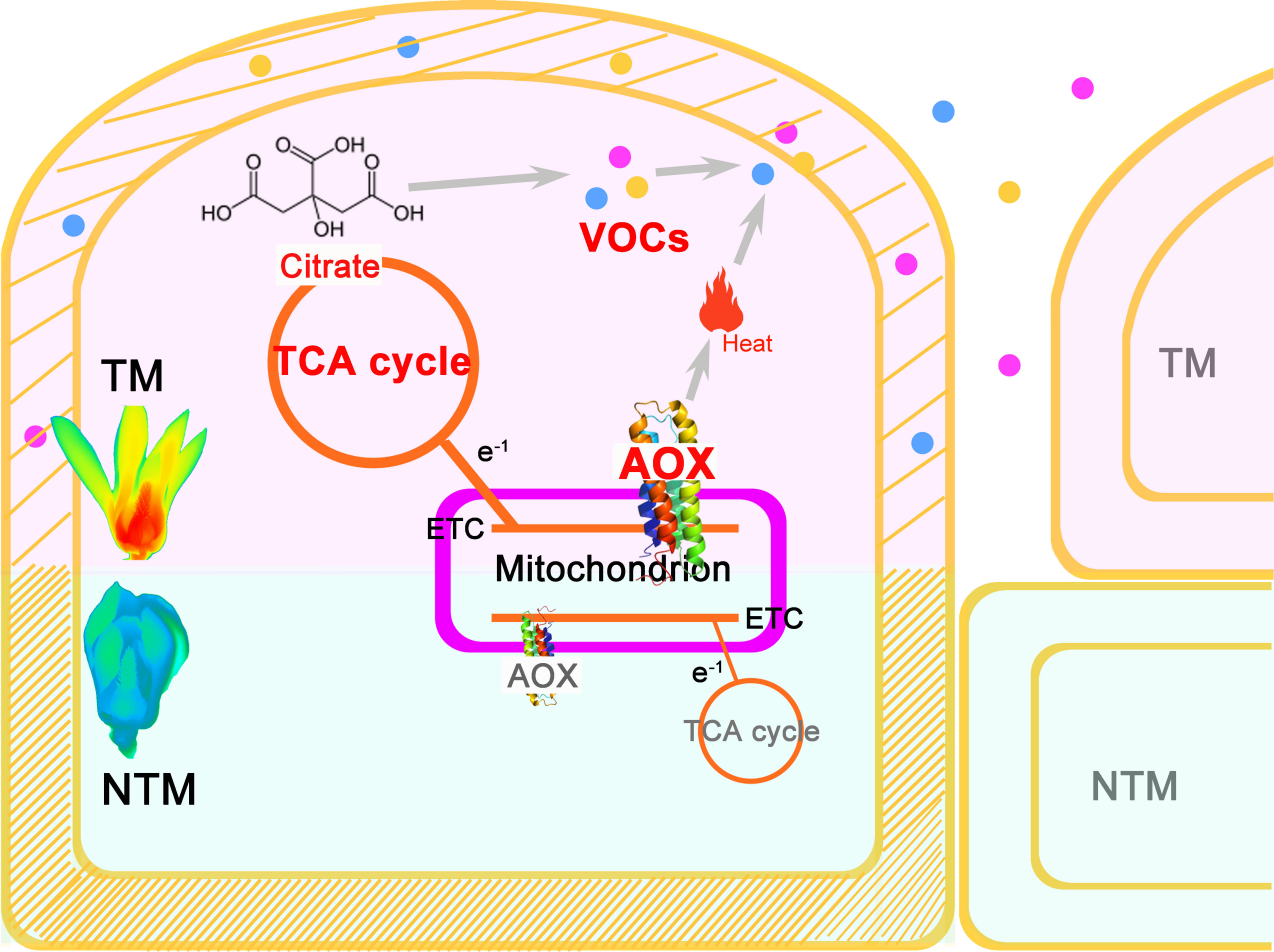
A central role of AOX in the multifaceted and intertwined biological processes during floral thermogenesis of *M. denudata*. When an increase of AOX activity produces remarkable heat in the major themogenic tissue gynoecium, the flower enters the TM stage. Meanwhile, the increased activity of AOX respiration pathway leads to a higher flux rate of the TCA cycle, which supplies more citrate to floral VOC biosynthesis. Due to the heat produced by AOX activity and a change in the cell wall property, including a decrease in lignin content, floral VOCs get emitted from the gynocecium cells to the air. There is a structural change in the cells during thermogenesis, which results in more intercellular space and promotes VOC release. Thus, the AOX plays a central role in coupling heat production and floral odor production and emission in thermogenic plants.

In this study, we developed a roadmap for establishing the comprehensive regulatory framework of floral thermogenesis in *M. denudata* (**Figure 4**). AOX may play a central role in floral thermogenesis of *M. denudata*. Initially, an increase in activity of the AOX pathway appears to occur, leading to dissipation of energy as heat as well as an increase in TCA flux. This increased TCA flux provides essential metabolic skeletons for the biosynthesis of floral VOCs. On the other hand, heat produced by the AOX pathway may promote the emission of floral VOCs. Meanwhile, notable anatomical and cytological changes occur in the thermogenic tissue, including a decrease in lignin content in the cell wall and the enlargement of intercellular spaces. These structural changes may also facilitate the emission of floral VOCs.

## Data availability statement

The original contributions presented in the study are publicly available. This data can be found here: NCBI, SRP148509.

## Author contributions

RW conceived the project idea and designed the experiments. LC, YJ, LL, and LS performed the experiments. RW, YJL, and LC performed the data analysis and prepared the figures and tables. RW and YL advised on the analysis and interpretation of the results. RW prepared the manuscript. All authors contributed to the article and approved the submitted version.

## Funding

This work was supported by the Fundamental Research Funds for the Central Universities (No. 2021ZY63) and the National Natural Science Foundation of China (No. 31770201).

## Acknowledgments

The authors are grateful to Prof. Helping Cheng in Peking University, Prof. Reinhard Jetter in University of British Columbia and Dr. Dechang Cao for their insightful comments to the first draft. We would like to thank Ms. Fengqin Dong in the Institute of Botany, Chinese Academy of Sciences for her help in the experiment of SEM observation. Special thanks are due to Prof. Feng Peng and Ms. Chulan Zhang in Beijing Forestry University for their kind assistance in the Raman microscopic observation, and Dr. Miao Yu for his suggestions on the revision of figure. We thank National Center for Protein Sciences at Peking University in Beijing, China, particularly Guilan Li for assistance with Seahorse Extracellular Flux Analyzer.

## Conflict of interest

The authors declare that the research was conducted in the absence of any commercial or financial relationships that could be construed as a potential conflict of interest.

## Publisher’s note

All claims expressed in this article are solely those of the authors and do not necessarily represent those of their affiliated organizations, or those of the publisher, the editors and the reviewers. Any product that may be evaluated in this article, or claim that may be made by its manufacturer, is not guaranteed or endorsed by the publisher.

## Abbreviations

AOX, Alternative Oxidase; COX, cytochrome oxidation; DEG, differentially expressed genes; NTM, non-thermogenic; OCR, oxygen consumption rate; RNA-Seq, RNA-sequencing; TM, thermogenic; UCP, Uncoupling Protein; VOC, volatile organic compounds.
