# Supplementary figures and images for: Heat production and volatile biosynthesis are linked via alternative respiration in Magnolia denudata during floral thermogenesis
Source: Front Plant Sci. 2022 Oct 14;13:955665. doi: 10.3389/fpls.2022.955665 (PMC9614359; doi:10.3389/fpls.2022.955665)

**Additional file 5: Figure S1**. KOG categories of the transcriptome unigenes.


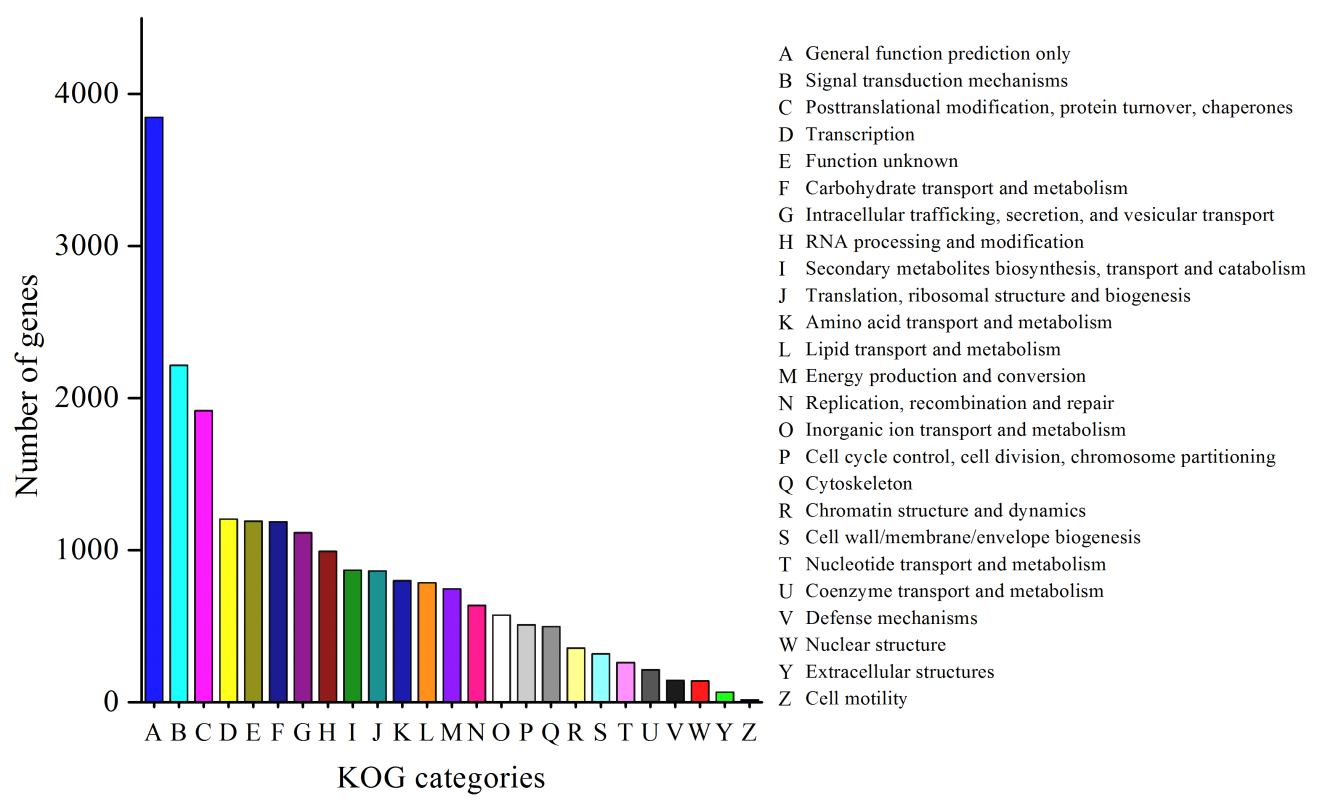

Supplement: Supplementary file 1 [file DataSheet_1.docx]

**Additional file 6: Figure S2**. PCA analysis of the four RNA-Seq samples.


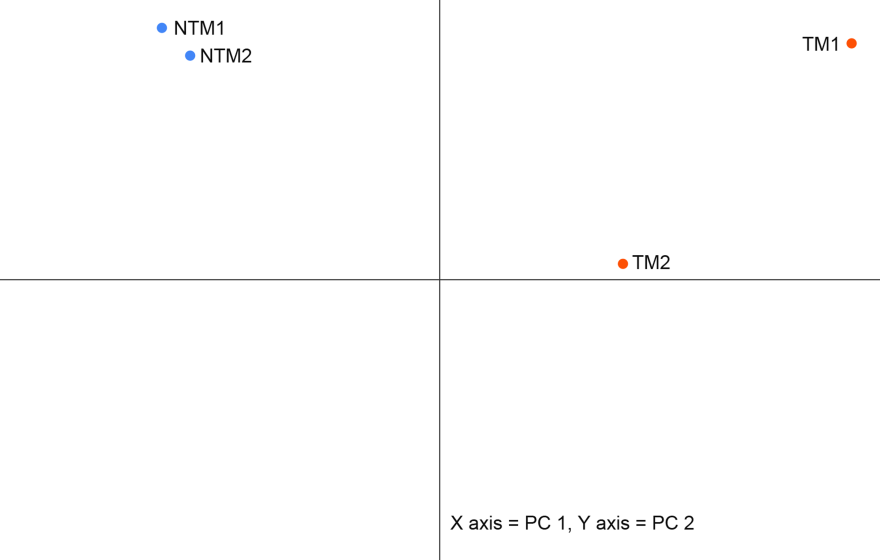

Supplement: Supplementary file 2 [file DataSheet_2.docx]
