# Supplementary material for: Heat production and volatile biosynthesis are linked via alternative respiration in Magnolia denudata during floral thermogenesis
Source: Front Plant Sci. 2022 Oct 14;13:955665. doi: 10.3389/fpls.2022.955665 (PMC9614359; doi:10.3389/fpls.2022.955665)
Supplement: Supplementary file 3 [file Table_1.docx]

**Additional file 1: Table S1.** Gene-specific primers for qRT-PCR

| Unigene | Primer | Sequence (5'->3') |
| --- | --- | --- |
| comp68798_c0_seq2 | F | GCGTGCCATCATCGGAAAAA |
|  | R | ATCGCTTTCTGTCCTGCCAA |
| comp69266_c0_seq1 | F | AGCGATAGCGTATGTGCAGT |
|  | R | ACCATTTCAGCCCACAGACC |
| comp69394_c0_seq1 | F | CTGCATACCCCCACATGCTA |
|  | R | CATCACTCATTCGGGACGCA |
| comp70002_c0_seq1 | F | GCACTCAAAGGCAAGGCAAA |
|  | R | AGCCGTCGATCTCACACATC |
| comp71644_c0_seq1 | F | CTCTGGTCACTGCCGATGTT |
|  | R | CATGTGGGTTACTCCGTGCT |
| comp72602_c0_seq1 | F | CATCACCCCAGTTCCAGGTTT |
|  | R | TTCTCTTGCCTGAGCTTGTCC |
| comp73416_c0_seq2 | F | TTCTGCTTTCACCTTGTCCCT |
|  | R | CCAGGAGAGGCGTTCATGTTT |
| comp74985_c0_seq2 | F | TTTCGACCACCTGTTACCCC |
|  | R | TTGAAGCGCACTGGGAAGAG |
| comp74991_c0_seq1 | F | TTGATGTGGGCATTCTCTCCA |
|  | R | AGCACCCAACAGATCCTTCTC |
| comp75001_c0_seq1 | F | CGTAAGCGAAGAAGGGGTAGG |
|  | R | TCTCTTCCCCAGTAGCCCTTT |
| comp76902_c3_seq1 | F | CCTTTCCTCATGGATACGGCT |
|  | R | CGTGGGGCTTGGAAAAGATTG |
| comp77614_c0_seq1 | F | AGAACCGCTCCAATTTCACCA |
|  | R | ACCACCCAAGAGAAATCGGTC |
| comp77670_c0_seq1 | F | AACGGGTCTTGAGGGGTTTT |
|  | R | GAAGAAAGCGAAAGGAGCGG |
| comp77715_c0_seq1 | F | CAAACACTGGTGGGAAGGAGA |
|  | R | AGCAACACGTCAGCAGTAGAA |
| comp73456_c0_seq2 | F | CTCAAGCGAACTCAACCCAAC |
|  | R | TCTTGAATCTTGGTGAGCGGT |
| GAPDH | F | GCAAGGATTGGAGAGGTGGAA |
|  | R | AGATCCACCACCGAAACATCC |
