# Supplementary material for: Heat production and volatile biosynthesis are linked via alternative respiration in Magnolia denudata during floral thermogenesis
Source: Front Plant Sci. 2022 Oct 14;13:955665. doi: 10.3389/fpls.2022.955665 (PMC9614359; doi:10.3389/fpls.2022.955665)
Supplement: Supplementary file 4 [file Table_2.docx]

**Additional file 2: Table S2**. Summary of the RNA-Seq raw data.

| Sample | Raw Data (Read) | Base | Q20% | Q30% | GC% |
| --- | --- | --- | --- | --- | --- |
| TM1 | 11805708 | 0.43G | 98.84 | 95.44 | 49.00 |
| TM2 | 11046638 | 0.40G | 99.50 | 98.07 | 47.74 |
| NTM1 | 10454549 | 0.52G | 98.72 | 95.33 | 46.36 |
| NTM2 | 7629400 | 0.38G | 98.58 | 95.81 | 46.36 |
