# Supplementary material for: Heat production and volatile biosynthesis are linked via alternative respiration in Magnolia denudata during floral thermogenesis
Source: Front Plant Sci. 2022 Oct 14;13:955665. doi: 10.3389/fpls.2022.955665 (PMC9614359; doi:10.3389/fpls.2022.955665)
Supplement: Supplementary file 5 [file Table_3.docx]

**Additional file 3: Table S3.** Cleaning of the RNA-Seq data.

|  | TM1 | | TM2 | | NTM1 | | NTM2 | | Total | |
| --- | --- | --- | --- | --- | --- | --- | --- | --- | --- | --- |
|  | Reads | % | Reads | % | Reads | % | Reads | % | Reads | % |
| Raw Data | 11805708 | 100.00 | 11046638 | 100.00 | 10454549 | 100.00 | 7629400 | 100.00 | 40936295 | 100.00 |
| After Adaptors Cut | 11798768 | 99.94 | 11040889 | 99.95 | 10419915 | 99.67 | 7624157 | 99.93 | 40883729 | 99.87 |
| After Junk filter | 11794474 | 99.90 | 11036336 | 99.91 | 10388012 | 99.36 | 7603089 | 99.66 | 40821911 | 99.72 |
| Valid Data | 11794474 | 99.90 | 11036336 | 99.91 | 10388012 | 99.36 | 7603089 | 99.66 | 40821911 | 99.72 |
