# Supplementary material for: Heat production and volatile biosynthesis are linked via alternative respiration in Magnolia denudata during floral thermogenesis
Source: Front Plant Sci. 2022 Oct 14;13:955665. doi: 10.3389/fpls.2022.955665 (PMC9614359; doi:10.3389/fpls.2022.955665)
Supplement: Supplementary file 6 [file Table_4.docx]

**Additional file 4: Table S4.** Summary of the transcriptome assembly.

|  | Transcripts | Unigenes |
| --- | --- | --- |
| Total number of reads | 73962 | 61212 |
| Minimum length of reads (bp) | 201 | 201 |
| Median length of reads (bp) | 469 | 406 |
| Average length of reads (bp) | 777 | 706 |
| Maximum length of reads (bp) | 1259 | 1154 |
| N_50_ (bp) | 7944 | 7944 |
| Total nucleotide length (bp) | 57528531 | 43275273 |
