# Supplementary material for: Heat production and volatile biosynthesis are linked via alternative respiration in Magnolia denudata during floral thermogenesis
Source: Front Plant Sci. 2022 Oct 14;13:955665. doi: 10.3389/fpls.2022.955665 (PMC9614359; doi:10.3389/fpls.2022.955665)
Supplement: Supplementary file 7 [file Table_5.docx]

**Additional file 5: Table S5**. Summary of the transcriptomic gene annotation.

|  | Number of annotated genes | Percentage of annotated genes |
| --- | --- | --- |
| NR | 35033 | 57.23 |
| Swiss-Prot | 21679 | 35.42 |
| Pfam | 25595 | 41.81 |
| GO | 19045 | 31.11 |
| KEGG | 14194 | 23.19 |
| KOG | 19069 | 31.15 |
| Total | 61212 | 100 |
