# Supplementary material for: Heat production and volatile biosynthesis are linked via alternative respiration in Magnolia denudata during floral thermogenesis
Source: Front Plant Sci. 2022 Oct 14;13:955665. doi: 10.3389/fpls.2022.955665 (PMC9614359; doi:10.3389/fpls.2022.955665)
Supplement: Supplementary file 8 [file Table_6.docx]

**Additional file 8: Table S6**. Enriched GO terms for the DEGs of RNA-Seq.

| **DEG group** | **GO term_ID** | **description** | **Log10 P value** | **Log2Odd ratio** | **Code color** |
| --- | --- | --- | --- | --- | --- |
| Group 1 | GO:0006259 | DNA metabolic process | -3.0655 | 0.997779492 | Cyan |
| Group 1 | GO:0006260 | DNA replication | -12.2924 | 2.194254397 | Cyan |
| Group 1 | GO:0022616 | DNA strand elongation | -1.8861 | 2.910843855 | Cyan |
| Group 1 | GO:0051174 | regulation of phosphorus metabolic process | -4.1192 | 2.385888664 | Cyan |
| Group 1 | GO:0051338 | regulation of transferase activity | -5.0132 | 2.595342029 | Cyan |
| Group 1 | GO:0065009 | regulation of molecular function | -2.3098 | 1.580922969 | Cyan |
| Group 1 | GO:0070271 | protein complex biogenesis | -8.1871 | 1.815622674 | Cyan |
| Group 1 | GO:0071103 | DNA conformation change | -20.8539 | 3.103488933 | Cyan |
| Group 1 | GO:0071824 | protein-DNA complex subunit organization | -11.0969 | 3.018600035 | Cyan |
| Group 1 | GO:0006928 | movement of cell or subcellular component | -5.7696 | 1.859931901 | Red |
| Group 1 | GO:0007017 | microtubule-based process | -7.699 | 1.936710487 | Red |
| Group 1 | GO:0007018 | microtubule-based movement | -9.1739 | 2.317613738 | Red |
| Group 1 | GO:0009812 | flavonoid metabolic process | -1.4559 | 1.933563931 | Green |
| Group 1 | GO:0009813 | flavonoid biosynthetic process | -2.041 | 2.123666815 | Green |
| Group 1 | GO:0007049 | cell cycle | -14.3188 | 1.626675236 | Blue |
| Group 1 | GO:0022402 | cell cycle process | -9.8239 | 1.747345123 | Blue |
| Group 1 | GO:0051301 | cell division | -8.1308 | 1.613163306 | Blue |
| Group 3 | GO:0043900 | regulation of multi-organism process | -2.6778 | 2.618108588 | Yellow |
| Group 3 | GO:0019748 | secondary metabolic process | -2.0757 | 1.463048936 | Yellow |
| Group 3 | GO:0044038 | cell wall macromolecule biosynthetic process | -5.1135 | 2.275220874 | Cyan |
| Group 3 | GO:0042546 | cell wall biogenesis | -7.3279 | 2.454952634 | Cyan |
| Group 3 | GO:0046274 | lignin catabolic process | -6.6021 | 3.224889199 | Cyan |
| Group 3 | GO:0071554 | cell wall organization or biogenesis | -5.9208 | 1.695552673 | Cyan |
| Group 3 | GO:0080051 | cutin transport | -4.585 | 4.001655801 | Cyan |
| Group 3 | GO:0006022 | aminoglycan metabolic process | -6.8861 | 3.139159325 | Cyan |
| Group 3 | GO:0006040 | amino sugar metabolic process | -1.5229 | 2.760647702 | Cyan |
| Group 3 | GO:0009611 | response to wounding | -6.5376 | 2.106244702 | Magenta |
| Group 3 | GO:0009635 | response to herbicide | -1.9208 | 3.653732498 | Magenta |
| Group 3 | GO:0032101 | regulation of response to external stimulus | -7.5229 | 3.594838809 | Magenta |
| Group 3 | GO:0001101 | response to acid chemical | -4.0862 | 1.062964375 | Magenta |
| Group 3 | GO:0006952 | defense response | -3.8539 | 1.006383098 | Magenta |
| Group 3 | GO:0031407 | oxylipin metabolic process | -1.7959 | 2.466105495 | Magenta |
| Group 3 | GO:0031408 | oxylipin biosynthetic process | -1.7959 | 2.466105495 | Magenta |
| Group 3 | GO:0009911 | positive regulation of flower development | -2.3979 | 2.694374482 | Blue |
| Group 3 | GO:0010047 | fruit dehiscence | -4.7447 | 3.746841902 | Blue |
| Group 3 | GO:0007568 | aging | -2.1612 | 1.653732498 | Blue |
| Group 3 | GO:0010260 | animal organ senescence | -2 | 1.937525464 | Blue |
| Group 3 | GO:0009838 | abscission | -1.3768 | 2.300095543 | Blue |
| Group 3 | GO:0008285 | negative regulation of cell proliferation | -1.699 | 2.609338378 | Blue |
| Group 3 | GO:0009435 | NAD biosynthetic process | -3.1249 | 3.46108742 | Green |
| Group 3 | GO:0043269 | regulation of ion transport | -2.1367 | 2.795751503 | Red |
